# A survey of knowledge, perceptions and use of core outcome sets among clinical trialists

**DOI:** 10.1186/s13063-021-05891-5

**Published:** 2021-12-19

**Authors:** Chiara Bellucci, Karen Hughes, Elaine Toomey, Paula R. Williamson, Karen Matvienko-Sikar

**Affiliations:** 1 School of Public Health, University College Cork, Cork, Ireland; 2 MRC Hub for Trials Methodology Research Network, Department of Biostatistics, University of Liverpool, Liverpool, UK; 3 School of Allied Health, University of Limerick, Limerick, Ireland; 4 MRC/NIHR Trials Methodology Research Partnership, Department of Biostatistics, University of Liverpool, Liverpool, UK

**Keywords:** Core outcome sets, Trials, Knowledge, Attitudes, Uptake

## Abstract

**Background:**

Core outcome sets (COS) are standardised sets of outcomes, which represent the minimum outcomes that should be measured and reported in clinical trials. COS can enhance comparability across health trials by reducing heterogeneity of outcome measurement and reporting and potentially minimising selective outcome reporting. Examining what researchers involved in trials know and think about COS is essential to increase awareness and promote COS uptake. The aim of this study is therefore to examine clinical trialists’ knowledge, perceptions and experiences of COS.

**Methods:**

An online survey design was used. Participants were clinical trialists, operationalised for the current study as researchers named as the contact person on a trial registered on the International Standard Randomised Controlled Trial Number (ISRCTN) Trial repository between 1 January 2019 and 21 July 2020. Survey items assessed clinical trialists’ familiarity with and understanding of COS, along with experiences of COS use and development.

**Results:**

Of 1913 clinical trialists contacted to participate, 62 (3%) completed the survey. Forty (65%) participants were familiar with COS and, of those familiar with COS, 21 (55%) had been involved in a trial that used a COS. Of clinical trialists who used COS in a trial(s), less than half (*n* = 9, 41%) reported that all COS outcomes were used. The main barriers to using COS are poor knowledge about COS (*n* = 43, 69%) and difficulties identifying relevant COS (*n* = 42, 68%). Clinical trialists also reported perceptions of COS as restrictive and often containing too many outcomes. The main enablers to using COS are clear understanding (*n* = 51, 82%) and perceived importance of COS (*n* = 44, 71%).

**Conclusions:**

Enhancing clinical trialists’ use of all COS outcomes is needed to reduce outcome heterogeneity and enhance comparability across trial findings. Enhancing awareness of COS importance among researchers and funders is needed to ensure that COS are developed and used by clinical trialists. Education and training may further promote awareness and understanding of COS.

**Supplementary Information:**

The online version contains supplementary material available at 10.1186/s13063-021-05891-5.

## Introduction

Core outcome sets (COS) are defined as standardised sets of outcomes, which represent the minimum outcomes that should be measured and reported in clinical trials for a particular area of health or healthcare [1–3]. The use of COS helps to address important issues in trial design, conduct and reporting, including heterogeneity in outcome measurement and reporting, and selective outcome reporting [1]. Heterogeneity in outcomes measured and reported has been identified across trials in multiple areas of health research [4, 5]. Heterogeneity limits synthesis of intervention effects, which has significant implications for determining which interventions are most efficacious [3]. For instance, an examination of neonatal Cochrane reviews found that half of the reviewed studies were inconclusive, with heterogeneity of outcomes being the most significant contributor [6]. Selective outcome reporting involves researchers publishing only a subset of outcomes examined (often those with positive results, leading to outcome reporting bias). For instance, a review of 102 trials, including 3736 outcomes, found that 50% of efficacy and 65% of harms outcomes were incompletely reported; statistically significant outcomes were more likely to be reported than null results [7]. Selective outcome reporting is problematic because it limits transparency and interpretability of research findings, and un-published results from trials and evidence syntheses remain inaccessible to patients, the public and policy makers [8, 9]. As COS represent standardised sets of outcomes that are the minimum to be all measured across analogous research fields, they facilitate evidence synthesis [1, 2, 10] and reducing research waste [11, 12]. In addition, COS development involves incorporation of key stakeholder perspectives, which ensures inclusion of clinically important and relevant outcomes, and increases the likelihood of COS uptake [13–16].

Up until the end of 2019, 370 COS studies had been published, relating to 447 COS, with approximately 200 COS currently being developed [17]. COS have been developed in diverse health areas including anaesthesia and pain control, blood disorders, child health, dentistry and oral health, and mental health [18]. Development and use of COS is supported by the Core Outcome Measures in Effectiveness Trials (COMET) Initiative. The COMET initiate website (http://www.comet-initiative.org/) includes a user-friendly database of applied and methodological resources to enable the development, identification and uptake of COS. Published guidelines further support COS development and reporting, including the COMET Handbook (Version 1) [1], the Core Outcome Set-STAndards for Development (COS-STAD) [11], the Core Outcome Set STAndardised Protocol Statement (COS-STAP) [19], and the Core Outcome Set STAndards for Reporting (COS-STAR) [20]. Development of a COS involves a number of steps. In short, these include determination of the scope and need for a specific COS, including identification of potential overlap between existing COS [1, 11]. Literature reviews of quantitative and/or qualitative research are then used to identify existing outcomes; studies involving primary data collection can also be used to identify existing outcomes. Consensus processes with stakeholder groups, typically a Delphi study followed by a consensus meeting, are then conducted [1, 11]. Consideration of dissemination and uptake of COS is important throughout the process [1, 21]. This will maximise the likelihood that the COS will be of benefit to research by improving evidence syntheses and reducing minimising research waste; conversely, if COS are not used, they are of no benefit and may contribute to research waste [1, 21].

One area in which COS uptake has been well examined to date is rheumatoid arthritis, with uptake of rheumatoid arthritis COS within clinical trials increasing over time [12, 22]. Across other areas of health and healthcare research, COS uptake in trials is unclear [23]. Use of COS, or not, may be attributable to trialists’ perceptions of the relevance and scope of a particular COS for use in a trial [24]. Use of COS may also be predicated on perceived benefits and/or extrinsic motivations or requirements. For instance, research funders advocating for, or requiring, the use of COS may promote the uptake of COS by encouraging their use to those applying for funding [25]. Clinical trialists’ awareness, knowledge and opinions about COS are also likely to be significant factors influencing COS use. The importance of examining these factors has been highlighted in other areas of trials methodology such as intervention fidelity [26]. For instance, a recent study highlighted that poor knowledge and understanding of fidelity are key limitations to whether and how fidelity is addressed in trials [26]. The same may be true for whether and how COS are used in trials, and understanding trialists’ knowledge and understanding of COS could inform future approaches and resources to encourage COS uptake, including training. There is limited evidence for this however despite the importance of trials and trial findings for informing evidence syntheses and guiding healthcare decision making [27]. The aim of this study is therefore to examine clinical trialists’ knowledge, perceptions and experiences of COS.

## Methods

### Study design

An online survey design was used to examine clinical trialists’ knowledge, perceptions and experiences of COS.

### Participants

Participants in this study were researchers named as the contact person on a health care trial (clinical trialists), which was registered on the International Standard Randomised Controlled Trial Number (ISRCTN) Trial repository between 01 January 2019 and 21 July 2020. The ISRCTN registry is a primary clinical trial registry that recognises clinical research studies in progress or published, along with a unique identification number required for publication (https://www.isrctn.com). Participants in this study were required to be over 18 years of age, but there were no restrictions in regard to participant gender, geographical location or health area.

### Procedure

The ISRCTN registry was searched, and all trials published between 01 January 2019 and 21 July 2020 were identified. The following information was extracted for each trial by a single reviewer (CB) and checked by a second reviewer (KMS): name and surname of the main contact (clinical trialist) involved in the trial, clinical trialist’s email, title of the trial, ISRCTN trial registration number, country of residence of the clinical trialist and whether the trial was associated with COVID-19.

All clinical trialists named on the identified trials were contacted directly via email, with the exception of clinical trialists named on COVID-19 trials. COVID-19 related trials were excluded from the study as COS in relation to COVID-19 are being examined separately in collaboration with https://covid-evidence.org/. One thousand nine hundred thirteen clinical trialists were therefore emailed an invitation to participate in the survey, followed 3 weeks later by a reminder email if they had not yet completed the survey. Where automatic ‘out-of-office’ replies were received for the original and/or reminder email, these trialists were followed up separately. Both the initial invitation and the reminder email included an information leaflet and a link to the online survey. The online survey included the study information and a consent form, which participants read and completed prior to commencing the survey.

### Survey questions

The survey used was developed for the purposes of this study and included 29 closed questions and 8 open-ended questions. Questions and associated response options were developed by KMS and PRW, who have experience and expertise in COS studies, and were also informed by existing evidence from similar studies on COS (please see Supplementary File 1). Participants completed a different number of questions dependent on their familiarity with COS, whether they had been involved in development of a COS, and the degree of involvement in COS development. All participants answered questions about demographic characteristics, their familiarity with COS, and perceived barriers, facilitators and benefits of COS. In addition, participants familiar with COS answered questions on their knowledge and perceptions of COS, whether they were involved in a trial that used COS and whether they had ever been involved in the development of a COS. Participants who had been involved in a trial using a COS were asked about the degree of COS use and their experience of use; participants who had not been involved in a trial using a COS were asked if a search for a COS had been conducted. Participants who had been involved in development of a COS were also asked about the capacity of their development, including perceived barriers and enablers to COS development.

#### Demographics

The first five questions were answered by all participants and related to clinical trialists’ demographic details: country of residence, highest qualification, area of research, years of research experience, and years of research experience specific to trials.

#### Familiarity with COS

One question, answered by all participants, then asked about clinical trialists’ familiarity with COS. Clinical trialists familiar with COS were first asked how they became familiar with COS via a multiple choice question, including response options such as “I have attended a conference presentation/seminar/talk on core outcome sets”. If participants indicated that they were familiar with COS, they were also asked about their awareness and knowledge of COS, use of COS in trials and COS development.

#### COS knowledge and perceptions

Participants familiar with COS also responded to nine statements assessing their knowledge and perceptions of COS. Statements included “core outcome sets can involve input from relevant stakeholders” and were rated via a five point Likert scale, ranging from 1 (“strongly disagree”) to 5 (“strongly agree”). Participants then indicated their level of understanding (“How well would you describe your understanding of what core outcome sets are”) and perceived importance of COS (“How important do you think core outcome sets are in clinical trials”).

#### COS use in previous trials

Clinical trialists familiar with COSs were also asked whether they were involved in a trial that used COS. If participants answered “yes”, they were asked how many trials they were involved in that had used a COS, the area of research of the trial(s) in which a COS was used, if all COS outcomes had been measured, and their experience of using COS in trials. Participants who answered “no” (had not used COS in a trial) were asked if a search for COS was conducted for the trial(s) they were involved in and if not, why.

#### COS development

All participants who were familiar with COS (irrespective of use of COS in trials) were then asked if they had ever been involved in the development of a COS. If participants were involved in development of a COS, they indicated in what capacity they were involved in development (“Member of core outcome set development team” or “Core outcome set participant”), and the area of research for which the COS was developed. Participants who were involved in COS development were also asked about the barriers and enablers related to the development of COS, using multiple response options with no limit to the number of options participants could select.

#### Perceived barriers, facilitators and benefits to COS use

The final section of the survey included three multiple-response option questions and was answered by all participants. The first of these questions asked about perceived barriers to COS use (e.g. “poor knowledge about core outcome sets”). The second question asked about perceived enablers (e.g. “perceived advantages for design of new studies”). The third question asked about perceived benefits of COS use (e.g. “Standardisation of outcome reporting”). A final question invited participants to provide any additional comments regarding COS.

### Analysis

Data were descriptively analysed using SPSS statistics version 26. Continuous variables were not normally distributed and so medians, ranges and interquartile ranges were derived using descriptive analysis. Frequencies of categorical variables were derived using descriptive analysis. Quantitative findings are presented narratively and as both tables and figures. Open-ended questions were analysed using a thematic analysis approach (28), whereby participant responses were read and line-coded independently by two researchers (CB; KMS). Line codes were then developed into categories and themes using a constant comparative approach (28) by two researchers (CB; KMS).

## Results

Sixty-two (3%) of the 1913 trialists contacted completed the online survey. The majority of participants were from the UK (53%; *n* = 33) and had completed a PhD (55%; *n* = 34). The most commonly reported areas of research were public health (27%; *n* = 17) and rehabilitation (19%; *n* = 12). Participants reported a broad range of years of research experience (range = 2–35 years, median = 12.5 years) and experience specific to health trials (range = 0–30 years, median = 7.5 years). Participant characteristics are presented in Table 1 and Supplementary File 2.

**Table 1:** Participant characteristics (*n* = 62)

	***N*** (%)	
Country*		
UK	33 (53.2)	
Europe	16 (25.8)	
Asia	7 (11.3)	
North America	4 (6.5)	
Australia	1 (1.6)	
South America	1 (1.6)	
**Qualification**		
Undergraduate degree	4 (6.5)	
Masters	11 (17.7)	
MD	12 (19.4)	
PhD	34 (54.8)	
DSc	1 (1.6)	
Undergraduate degree	4 (6.5)	
**Area of research**		
Anaesthesia and pain control	2 (3.2)	
Blood disorders	2 (3.2)	
Cancer	5 (8.1)	
Child health	8 (12.9)	
Developmental, psychosocial and learning problems	2 (3.2)	
Ear, nose and throat	2 (3.2)	
Effective practice/health systems	1 (1.6)	
Endocrine and metabolic	3 (4.8)	
Eyes and vision	1 (1.6)	
Gastroenterology	3 (4.8)	
Gynaecology	1 (1.6)	
Health care of older people	8 (12.9)	
Heart and circulation	5 (8.1)	
Infectious disease	5 (8.1)	
Kidney disease	1 (1.6)	
Lungs and airways	7 (11.3)	
Mental Health	8 (12.9)	
Methodological and diagnostic	2 (3.2)	
Muscle disease	2 (3.2)	
Neonatal care	2 (3.2)	
Neurology	8 (12.9)	
Orthopaedics and trauma	3 (4.8)	
Pregnancy and childbirth	4 (6.5)	
Public health	17 (27.4)	
Radiology	2 (3.2)	
Rehabilitation	12 (19.4)	
Rheumatology	1 (1.6)	
Skin	2 (3.2)	
Tobacco, drugs and alcohol dependence	1 (1.6)	
Urology	1 (1.6)	
Wounds	2 (3.2)	
Other **	15 (24.2)	
	**M (SD)**	**Range**
Years of research experience	13.55 (8.38)	2–35
Years of research experience specific to health and/or healthcare trials	9.88 (7.81)	0–30

*M* mean; *SD* standard deviation

* Full details of participant country are presented in Supplementary File 2

** Health areas self-reported by participants, not listed in the COMET health areas; see Supplementary File 2

### Awareness, understanding and perceptions of COS

Participants’ awareness of COS, in terms of whether they are familiar with COS and how they became familiar with COS, is presented in Table 2. The majority of participants (65%; *n* = 40) were familiar with COS, while 22 participants (35.5%) were not familiar with COS. Of participants who were familiar with COS, the majority had seen a COS reported in a trial (60%, *n* = 24) or other type of research (63%; *n* = 25); 53% had used a COS (*n* = 21). The majority had not developed nor participated in development of a COS (neither 73%, *n* = 29). The majority had also not received any academic education about COS (85%, *n* = 34) or attended any external training on COS (90%, *n* = 34).

**Table 2:** Participant awareness of core outcome sets (COS) for participants familiar with COS (*n* = 40, 64.5%)

	***N*** (%)	
	Yes	No
Have used a COS	21 (52.5)	19 (47.5)
Have developed a COS	10 (25)	20 (75)
Have seen a COS reported in a trial	24 (60)	16 (40)
Have seen a COS reported/discussed in another type of research (e.g. evidence synthesis)	25 (62.5)	15 (37.5)
Participated in the development of a COS as a participant/stakeholder	11 (27.5)	27 (72.5)
Received education on COS as part of an academic course	6 (15)	34 (85)
Attended training on COSs (external to academic coursework)	4 (10)	36 (90)
Attended a conference presentation/seminar/talk on COS	15 (37.5)	23 (62.5)
Informed about COS by colleague	8 (20)	32 (80)
Funded a COS*	1 (2.5%)	39 (97.5)
Applied to develop a COS*	1 (2.5%)	39 (97.5)

Note: * = self-reported by participants

Clinical trialists’ understanding of COS is presented in Table 3. Overall participants’ responses reflected good understanding of COS; participants’ median self-reported understanding was 4 (range 2–5) on the 5-point scale with higher scores indicative of greater understanding. Participants also reported high perceived importance of COS in clinical trials (median = 3, range = 2–3) measured on a 3-point scale.

**Table 3:** Trialists’ understanding of core outcome sets (COS)

	Median	Range	IQR
COS are the minimum that should be ***measured*** for specific health or health care area	4	1–5	4
COS are the minimum that should be ***reported*** for specific health or health care area	4	1–5	4
All outcomes in the COS should be measured	4	1–5	2
Other outcomes can be measured in addition to outcomes in COS	5	2–5	1
COS can be used in research other than trials (e.g. evidence synthesis, observational studies)	5	3–5	1
COS are relevant to clinical audit and routine care	4	3–5	1
COS can involve input from relevant stakeholders	5	3–5	1
COS require consensus processes in development	5	2–5	1
Development of a COS involves multiple stages	5	4–5	0.75
Understanding of what COS are	4	2–5	1
Perceived importance of COS in clinical trials	3	2–3	1

*IQR* interquartile range

The last question is measured on a scale of 1 (not important) to 3 (very important); all other questions are measured using a scale from 1 (strongly disagree) to 5 (strongly agree); only participants who reported being familiar with COS (*n* = 40) responded to these questions

**Fig. 1:**
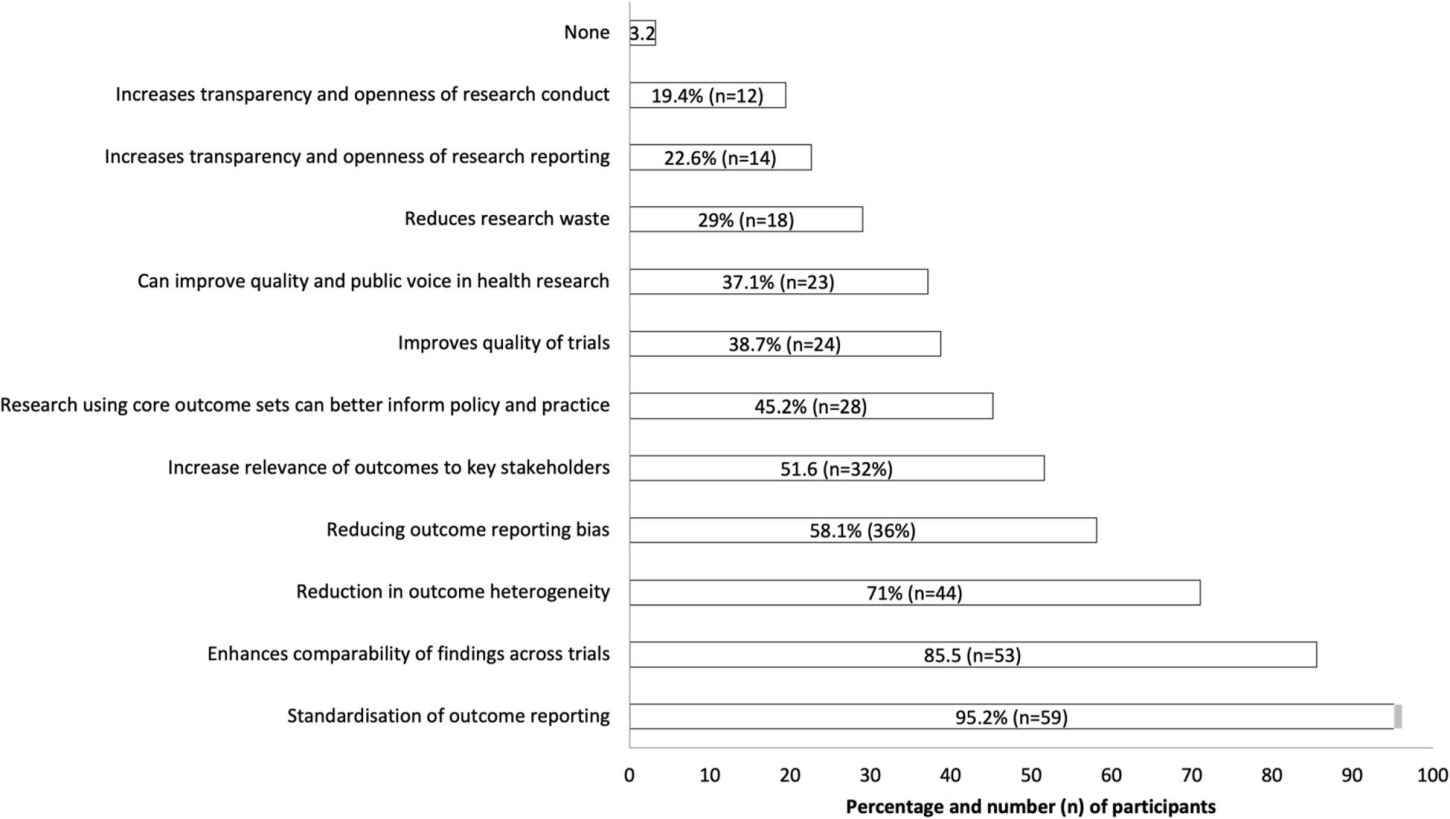
Trialist perceptions of potential benefits of using COS

Participants’ perceptions of potential benefits of using COS are presented in Fig. 1. Nearly all participants reported that they think COS can improve standardisation of outcome reporting (96%, *n* = 53) and enhance comparability of findings across trials (86%, *n* = 53). Only ten participants (16%) thought that COS can enhance the patients’ and public voice in research (i.e. the amount of patient input and involvement); while 37% thought it could improve the *quality* of the public voice in research. Similarly, only twelve participants (19%) and 14 participants (23%) felt that COS can improve transparency and openness of research conduct and reporting respectively. Two participants (3.2%) reported no benefits of using COS in trials.

### Use of core outcome sets in clinical trials

Of the 40 participants who were familiar with COS, 21 participants (55%) reported being involved in a trial that used a COS; 15 participants (39.5%) reported not being involved in a trial using a COS, while four participants did not respond. See Supplementary File 3 for the health area for which COS was used in a trial. Trialists most commonly reported being involved in one trial that used a COS (*n* = 12 participants); see Table 4. Less than half of participants reported that all COS outcomes were used in the trial they were involved in (41%; *n* = 9). In response to an open-ended question, clinical trialists who did not measure all COS outcomes in the trial they were involved in provided the following reasons for not using all outcomes: (1) an outcome was excluded due to how it was measured, (2) lack of COS specific to the intervention and population/focus of the study, (3) due to the time limit of the specific trial a COS outcome was deemed not essential, (4) unspecified impact of COVID-19 on trial conduct and (5) due to lack of validated measures for the COS outcomes. The most common sources for identifying outcomes in trials overall were outcomes used in previous trials (82%, *n* = 18), patient and public involvement (68%, *n* = 15), and practitioner opinion (68%, *n* = 15).

**Table 4:** Use of COS in trials

	Yes	No	Don’t know
	***N*** (%)	***N*** (%)	***N*** (%)
Involved in a trial that used a COS	21 (55.3)	15 (39.5)	–
All COS outcomes used in trial	9 (42.9)	10 (47.6)	2 (9.5)
Search conducted to identify COS for use in trial^a^	6 (15.8)	7 (18.4)	–
**Sources used to identify other trial outcomes**			
Patient and public involvement	15 (71.40)	6 (28.6)	
Practitioner opinion	15 (71.40)	6 (28.6)	
Outcomes used in other trials	17 (81)	4 (19)	
Recommendations from a professional body	11 (52.4)	10 (47.6)	
Recommendations from a funding body	0	21 (100)	
Information from a feasibility/pilot study	9 (42.9)	12 (57.1)	
Consensus process among all colleagues involved^b^	1 (4.76)	20 (95.24)	
Personal experience and expertise^b^	1 (4.76)	20 (95.24)	
Aim of the intervention^b^	1( 4.76)	20 (95.24)	
	**Median**	**Range**	**IQR**
Number of trials involved in that used a COS	1	1–10	2.25

36 trialists, who had previously reported familiarity with COS, provided data on COS use in trials

*IQR* interquartile range

^a^Question asked to participants who reported not being involved in a trial that used a core outcome set (*n* = 15)

^b^Sources self-reported by participants

### Barriers, enablers, benefits and general opinions of all clinical trialists

**Fig. 2:**
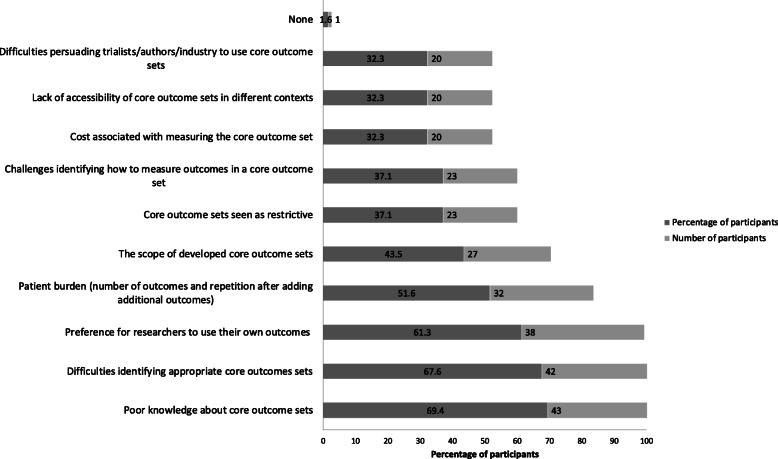
Barriers to COS use

**Fig. 3:**
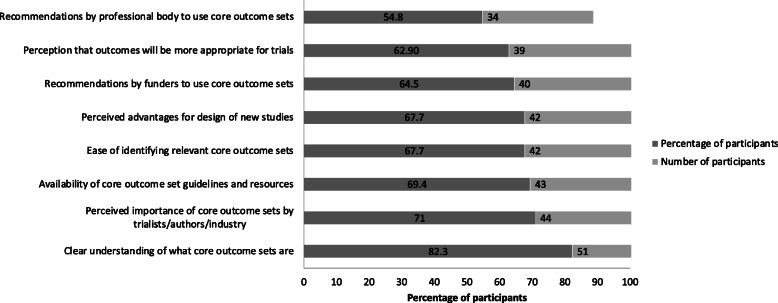
Enablers for COS use

Perceived barriers and enablers to COS use are presented in Fig. 2 and Fig. 3 respectively. The most commonly endorsed barriers to COS use were poor knowledge about COS (reported by 69%, *n* = 43), difficulties identifying appropriate COS (reported by 68%, *n* = 42) and researchers’ preference to use their own choice of outcomes (61%, *n* = 38). The most commonly endorsed enablers to COS use were clear understanding of what COS are (reported by 82%, *n* = 51), perceived importance of COS by clinical trialists, authors/industry (reported by 71%, *n* = 44) and availability of COS guidelines and resources (reported by 69%, *n* = 43).

### Development of COS by clinical trialists

Eighteen participants reported being involved in development of a COS, with half (*n* = 9, 50%) reporting they were involved in a COS development team and half (*n* = 9, 50%) reporting they were a participant in a COS development process. Twenty participants reported not being involved in development of a COS. See Supplementary File 4.

**Fig. 4:**
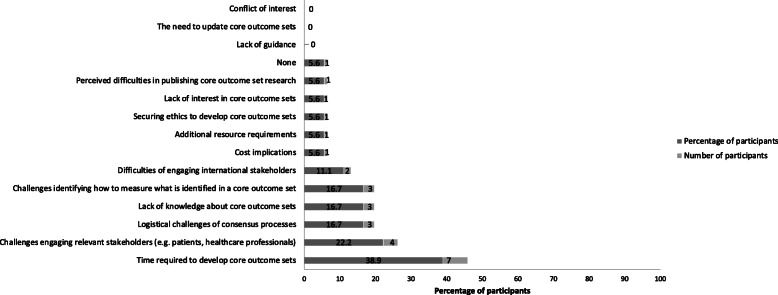
Barriers to COS development

**Fig. 5:**
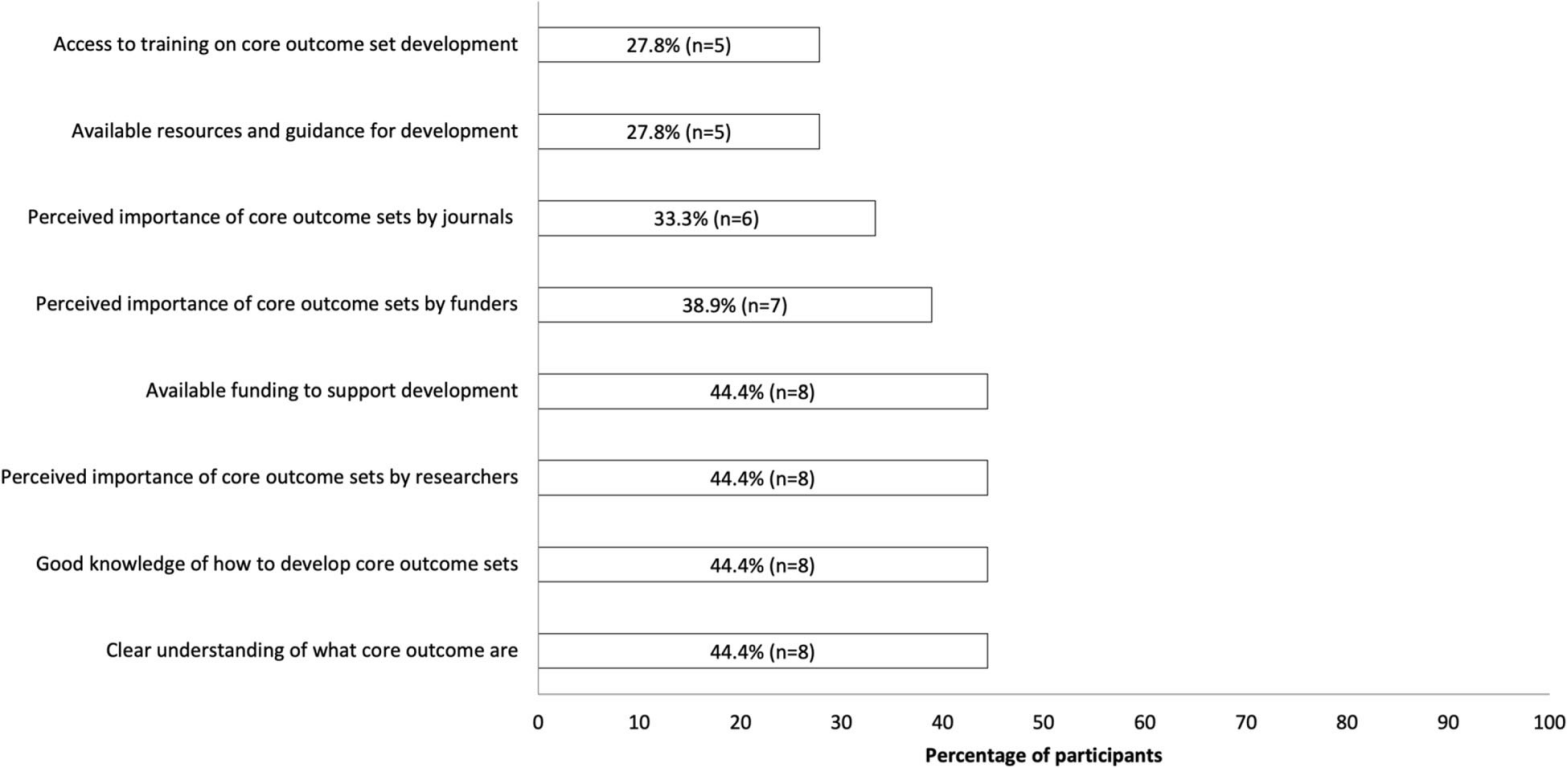
Enablers COS development

Perceived barriers to COS development are presented in Fig. 4. The most common barriers to developing COS were the time required to develop COS (reported by 39%, *n* = 7) and challenges engaging relevant stakeholders (reported by 22%, *n* = 4). Perceived enablers to COS development are presented in Fig. 5. The most commonly reported enablers to developing COS were a clear of understanding of what COS are, how to develop COS, perceived importance by researchers and available funding to support development (all reported by 44%, *n* = 8).

### Clinical trialists’ general perceptions of COS

Thirteen participants also provided responses to an open-ended question about their thoughts on COS. Five themes were identified, which include “Too many outcomes in COS”, “Specificity of COS”, “Restrictiveness”, “Impact on trials” and “Strategies to Enhance Knowledge and Use”. The first theme was identified from the responses of five participants who felt that an important barrier to COS use is in relation to a COS containing too many outcomes. Participants felt that having too many outcomes in COS is impractical and could result in researchers omitting other important outcomes, as well as limiting ability to report all outcomes, and increasing burden for researchers, particularly in cohort studies where multiple outcomes are collected. For example, “Although I find core outcome sets useful in theory- when these sets include 100’s of “core” outcomes its often not practical to include them all”. In terms of specificity of COS, four participants felt there was a need for greater specificity in COS as they are not specific enough for clinical trials. This includes in relation to the use of COS in different contexts or that there may be multiple potentially relevant COS for any given trial. For example, “Would be concerned about having to sacrifice disease-specific outcomes to accommodate core, but potentially less relevant, outcomes”.

The theme of restrictiveness, which was reported by two clinical trialists, related to perceptions of COS as restrictive and potentially limiting to innovation. For instance, it was felt that using COS would limit ability to explore and examine new or different outcomes in trials; “I believe having COS per trial scope will limit the innovation of new outcomes or test them out”. The theme Impact on Trials includes both negative and positive aspects of COS use in trials. Two participants felt that COS can negatively impact research and trial processes, such as increasing researcher and participant burden in relation to data collection. For example, “one study may have multiple relevant core outcome sets as they can exist for fields, interventions and populations. I’m not sure how we’re meant to manage all those possibilities, especially when the priority for an individual study will be to choose the outcomes that best capture the effect of that specific intervention, and minimising the burden of too many questionnaires to encourage participation”. One participant did report perceived benefits of COS for improving aspects of research such as synthesis and trial processes; “I can see the advantages for pooling data, particularly in areas where recruitment is challenging”.

Strategies to enhance COS knowledge and use were reported by two participants. They noted the importance of collaboration and buy-in from the research community, including internationally, to further knowledge and COS use. The importance of education and training to support COS use was also reported. “Buy in from research community via education/training is important (especially for junior researchers). But, requirements by funders is essential (unfortunately), same goes for clinical trialists reviewers.”

## Discussion

This study is the first examination of the knowledge, perceptions and use of COS among a general sample of clinical trialists involved in health care trials registered on a clinical trial registry. Our findings indicate good awareness and use of COS among our sample of respondents. Overall, the main barriers to COS use related to knowledge about COS and difficulties identifying relevant COS, while the main enablers were a clear understanding and the perceived importance of COS. Involvement in COS development as either a developer or participant was moderate among clinical trialists in our study. Identified barriers to COS development included the time involved in development and difficulties engaging stakeholders; enablers include clear understanding of what COS are and how to develop them, perceived importance of COS and availability of funding.

The majority of clinical trialists in our study reported awareness of COS, which is promising as awareness of COS is suggested to increase COS uptake [28]. The most common source of COS awareness in the current study was seeing COS reported in trials or other types of research, such as systematic reviews. One third of clinical trialists familiar with COS had also attended a conference presentation, seminar and/or talk on COS. To date, 370 COS studies relating to 447 COS have been published [17], and both COS dissemination via presentations and publications are recommended strategies to improve COS uptake and implementation [1]. As such, our findings are promising because they demonstrate exposure of clinical trialists to COS in these forms of dissemination. Few clinical trialists in the current study reported receiving or attending any form of academic or professional education or training on COS. This is similar to findings from a survey of clinical trialists in the area of COS for hip fracture, who reported a need for increased research training to increase COS awareness and use [28]. The role of COS education and training is linked to clinical trialists’ perceptions of the importance of knowledge and understanding about COS. Clinical trialists in the current study reported a good overall understanding of COS, how they are developed and used and benefits of use, as well as highly endorsing the importance of COS for trials. Despite high levels of understanding and perceived benefits however, just over half of the clinical trialists familiar with COS in the current study reported using COS, though this may be attributable also to perceptions of COS as not being applicable to their trial area and/or a relevant COS not existing for their trial. Clinical trialists reported that while poor knowledge about COS is the greatest barrier to their use, having a clear understanding about what COS are is the greatest enabler. Similarly, knowledge about what COS are and how to develop COS were noted as important factors for COS development. As such, these findings taken together emphasise the importance of future awareness-raising initiatives, including COS training and education for all clinical trialists.

Information and awareness alone are insufficient to increase COS uptake, as indicated by behaviour change literature [29, 30] and additional barriers and enablers were reported by clinical trialists in this study. One barrier to COS use reported by clinical trialists was researcher preference to use their own outcomes, which is problematic when those outcomes are not those identified as essential by relevant stakeholders and are not comparable with outcomes in other trials. The ease with which clinical trialists are able to identify relevant COS was also noted as an important factor. Work is on-going to develop a guide for identification, selection and application of a relevant COS, which will function to support clinical trialists and minimise this barrier. This guide will complement existing guidance on COS development and reporting [1, 11, 19, 20] and relates to reported importance of the availability of COS guidelines and resources as an enabler to COS use in this study. Our finding that recommendations from funders to use COS is an important enabler is in line with findings from a previous survey, which noted that funding bodies can encourage clinical trialists to search for COS at the funding application stage [25]. A further barrier to COS use reported by clinical trialists included perceived patient and researcher burden when COS are perceived to contain a large number of outcomes and/or when additional outcomes are to be included in the trials. Perceived burden may lead to clinical trialists not including all COS outcomes and less than half of clinical trialists in the current study reported that all COS outcomes were used in the trial they were involved in. Other reasons for not including all outcomes included perceived relevance of outcomes, as has been found previously in relation to use of COS for rheumatoid arthritis [31], and measurement related issues. The development and identification of appropriate COS measurement tools is outlined and supported by the COSMIN and COMET initiatives [32], and a recent review demonstrated that an increasing number of studies are examining both the *how* (i.e. measurement) in addition to the *what* (the COS itself) [33]. This review also demonstrated that methods for selection of outcome measurement have improved since publication of the COSMIN and COMET guideline [32]. However, issues with perceived relevance, burden and measurement of COS are important considerations for COS future research and uptake in practice.

In relation to COS development by clinical trialists, few clinical trialists in the current study had been involved in the development of a COS. Previous research has suggested that perceptions of COS development as complex and resource intensive, adversely impact on COS development [31]. This is supported by our finding that clinical trialists reported the time required to develop COS as the greatest barrier. Difficulties engaging stakeholders was also noted in our study and has been noted elsewhere [21, 34], despite observed increases in stakeholder engagement in COS development [18]. For instance, previous interviews with COS developers indicated that engagement with stakeholders is seen as challenging in relation to their understanding and prioritisation of outcomes [34]. The OMERACT group similarly acknowledge challenges in engaging stakeholders and developed an integrated knowledge translation framework that provides guidance on stakeholder engagement [21]. This guidance includes identifying and engaging the ‘right’ stakeholders early in the process, and maximising involvement, using approaches such as virtual meetings [21]. The importance of available funding to develop COS as an enabler has also been found in previous interviews with COS developers [34]. This highlights the importance of trials methodology funding to support researchers to develop and use COS that can improve standardisation and comparative effectiveness health research.

It is important to note that, despite high levels of reported awareness and knowledge of COS in the current study, our sample is likely influenced by self-selection bias. Of the 1913 participants contacted to participate, only 62 (3%) took part. This suggests that participants who completed the survey may have been more likely to be familiar with COS anyway and/or to have a particular interest in outcome measurement and reporting in trials; this is further suggested by the high levels of understanding and perceived importance of COS in this study. Thus, our sample may be biased in that it includes those with existing knowledge and/or positive perceptions about COS, and our findings may not be generalisable beyond this group. Nevertheless, a strength of the current survey is the inclusion of a general sample of clinical trialists across health areas, without a focus on any one health area, which enabled a broader examination of clinical trialists’ awareness, knowledge and use of COS. Further, a previous survey with a comparable sample size identified some similar issues in the specific health area of hip-fracture [28], which suggests that engagement with COS surveys may be low more generally.

In conclusion, the majority of clinical trialists in this study were familiar with and had used COS, though this finding may reflect self-selection bias in our sample. The main barrier and enabler to COS development and use related to knowledge and understanding of what COS are and how to develop and use them. Coupled with a low level of reported education and training on COS, our findings indicate a need for greater education and promotion of awareness, understanding and uptake of COS among clinical trialists. There is also a need to enhance awareness of the importance of using all COS outcomes to ensure standardisation and comparability of trial outcomes. Greater awareness and use of COS by clinical trialists will be of benefit to comparative effectiveness research with potential for real and meaningful change in health research.

## Supplementary Information

**Additional file 1.** Core Outcome Set Survey.

**Additional file 2.** Full Participant Country and Health Area Characteristics.

**Additional file 3.** Use of COS in trials by health area.

**Additional file 4. **Trialist involved in the development of COS (*n*=18).

## Data Availability

The datasets used and/or analysed during the current study are available from the corresponding author on reasonable request.
