# Supplementary material for: A survey of knowledge, perceptions and use of core outcome sets among clinical trialists
Source: Trials. 2021 Dec 19;22:937. doi: 10.1186/s13063-021-05891-5 (PMC8684586; doi:10.1186/s13063-021-05891-5)
Supplement: Supplementary file 1 — Additional file 1. Core Outcome Set Survey. [file 13063_2021_5891_MOESM1_ESM.docx]

**Supplementary File 2: Core Outcome Set Survey**

**Demographics**

1. What country do you work in?
2. What is the highest qualification you have completed
   1. Undergraduate Degree
   2. Higher Diploma
   3. Masters Degree
   4. MD
   5. PhD
   6. Other:
3. What area(s) of research are you involved in (tick all that apply)
   1. Anaesthesia and pain control
   2. Blood disorders
   3. Cancer
   4. Child health
   5. Consumer & communication strategies
   6. Dentistry & oral health
   7. Developmental, psychosocial & learning problems
   8. Ear, nose & throat
   9. Effective practice/health systems
   10. Endocrine & metabolic
   11. Eyes & vision
   12. Gastroenterology
   13. Genetic disorders
   14. Gynaecology
   15. Health care of older people
   16. Heart & circulation
   17. Infectious disease
   18. Kidney disease
   19. Lungs and airways
   20. Mental health
   21. Methodological & diagnostic
   22. Muscle disease
   23. Neonatal care
   24. Neurology
   25. Orthopaedics & trauma
   26. Pregnancy & childbirth
   27. Public Health
   28. Radiology
   29. Rehabilitation
   30. Rheumatology
   31. Skin
   32. Tobacco, drugs & alcohol dependence
   33. Urology
   34. Wounds
   35. Other
4. How many years’ research experience do you have?
5. How many years of research experience do you have specific to trials related to health and/or healthcare?
6. Have you heard of/are you familiar with core outcome sets?
   1. Yes (go to question 7)
   2. No (go to question 23)
7. How did you hear about/become familiar with core outcome sets (tick all that apply)
   1. I have used a core outcome set(s)
   2. I have developed a core outcome set(s)
   3. I have seen a core outcome set reported in a trial
   4. I have seen a core outcome set reported/discussed in another type of research (e.g. evidence synthesis, core outcome set development paper)
   5. I participated in development of a core outcome set
   6. I received education on core outcome sets as part of an academic course
   7. I have attended training on core outcome sets (external to academic coursework)
   8. I have attended a conference presentation/seminar/talk on core outcome sets
   9. A colleague has told me about core outcome sets
   10. Other, please specify
8. How much do you agree with the following statements about core outcome sets (5 point scale)
   1. They are the minimum that should be measured for specific health or health care area
   2. They are the minimum that should be reported for specific health or health care area
   3. All outcomes in the core set should be measured
   4. Other outcomes can be measured in addition to outcomes in core outcome sets
   5. They can be used in research other than trials
   6. They are relevant to clinical audit and routine care
   7. Core outcome sets involve input from relevant stakeholders
   8. Core outcome sets require consensus processes in development
   9. Development of a core outcome set involves multiple stages
   10. None of the above
9. How well would you describe your understanding of what core outcome sets are? (1-5 scale)
10. How important do you think core outcome sets are in clinical trials?
    1. Not important, somewhat important, very important
11. Have you ever been involved in a trial that used a core outcome set?
    1. Yes (go to question 12)
    2. No (go to question 16)
12. How many trials that you have been involved in have used a core outcome set?
13. In what areas of research were you involved in a trial(s) that used a core outcome set (tick all that apply)?
    1. Anaesthesia and pain control
    2. Blood disorders
    3. Cancer
    4. Child health
    5. Consumer & communication strategies
    6. Dentistry & oral health
    7. Developmental, psychosocial & learning problems
    8. Ear, nose & throat
    9. Effective practice/health systems
    10. Endocrine & metabolic
    11. Eyes & vision
    12. Gastroenterology
    13. Genetic disorders
    14. Gynaecology
    15. Health care of older people
    16. Heart & circulation
    17. Infectious disease
    18. Kidney disease
    19. Lungs and airways
    20. Mental health
    21. Methodological & diagnostic
    22. Muscle disease
    23. Neonatal care
    24. Neurology
    25. Orthopaedics & trauma
    26. Pregnancy & childbirth
    27. Public Health
    28. Radiology
    29. Rehabilitation
    30. Rheumatology
    31. Skin
    32. Tobacco, drugs & alcohol dependence
    33. Urology
    34. Wounds
    35. Other

Were all outcomes in the core outcome set measured in the trial(s) you were involved in?

Yes

No, please specify why all outcomes were not measured

What did you find easy, or not, about using a core outcome set in the trial(s) you were involved in? [open ended response]

**[No I have not been involved in a trial that used a core outcome set]**

1. Was a search conducted to identify whether a core outcome set existed that could be used in the trial you were involved in?
   1. Yes (go to question 17)
   2. No (go to question 18)
   3. I don’t know
2. If a core outcome set could have been used in a trial you were involved in but was not, please indicate why [Open ended response]
3. Have you ever been involved in the development of a core outcome set
   1. Yes (go to question 19)
   2. No (go to question 23)
4. In what capacity were you involved in the development of a core outcome set?
   1. Member of core outcome set development team (go to question 20)
   2. Core outcome set participant (e.g. in consensus processes) (go to question 23)
5. For what area was your core outcome set developed (tick all that apply) [dropdown response option]?
   - Anaesthesia and pain control
   - Blood disorders
   - Cancer
   - Child health
   - Consumer & communication strategies
   - Dentistry & oral health
   - Developmental, psychosocial & learning problems
   - Ear, nose & throat
   - Effective practice/health systems
   - Endocrine & metabolic
   - Eyes & vision
   - Gastroenterology
   - Genetic disorders
   - Gynaecology
   - Health care of older people
   - Heart & circulation
   - Infectious disease
   - Kidney disease
   - Lungs and airways
   - Mental health
   - Methodological & diagnostic
   - Muscle disease
   - Neonatal care
   - Neurology
   - Orthopaedics & trauma
   - Pregnancy & childbirth
   - Public Health
   - Radiology
   - Rehabilitation
   - Rheumatology
   - Skin
   - Tobacco, drugs & alcohol dependence
   - Urology
   - Wounds
   - Other
6. Which, if any, of the following do you think are barriers to developing core outcome sets? (tick all that apply)
7. Challenges engaging relevant stakeholders (e.g. patients, healthcare professionals)
8. Cost implications
9. Time required to develop core outcome sets
10. Logistical challenges of consensus processes
11. Additional resource requirements, please specify
12. Difficulties of engaging international stakeholders
13. Securing funding to develop core outcome sets
14. Securing ethics to develop core outcome sets
15. Lack of guidance
16. Lack of interest in core outcome sets
17. Lack of knowledge about core outcome sets
18. The need to update core outcome sets over time
19. Conflict of interest
20. Perceived difficulties in publishing core outcome set research
21. Challenges identifying how to measure what is identified in a core outcomes set
22. None
23. Other, please specify
24. Which, if any, of the following do you think are enablers/facilitators to developing core outcome sets? (tick all that apply)
25. Clear understanding of what core outcome sets are
26. Good knowledge of how to develop core outcome sets
27. Perceived importance of core outcome sets by researchers
28. Perceived importance of core outcome sets by journals
29. Perceived importance of core outcome sets by funders
30. Available funding to support development
31. Available resources and guidance for development
32. Access to training on core outcome set development
33. None
34. Other, please specify
35. Which sources were used to identify outcomes in the trial you were involved in? (tick all that apply)
36. Patient and public opinion
37. Practitioner opinion
38. Outcomes used in other trials
39. Recommendation(s) from a professional body
40. Recommendation(s) from a funding body
41. Information from a feasibility/pilot study
42. Other, please specify

**If you are not familiar with Core Outcome Sets, they are defined as standardised minimum agreed upon sets of outcomes that should be measured and reported in all trials for a particular health area.**

1. Which, if any of the following do you think are barriers to using core outcome sets in trials? (tick all that apply)
2. Poor knowledge about core outcome sets
3. Difficulties persuading trialists/authors/industry to use core outcome sets
4. Difficulties identifying appropriate core outcomes set
5. Preference for researchers to use their own outcomes
6. The scope of developed core outcome sets
7. Core outcome sets seen as restrictive
8. Challenges identifying how to measure outcomes in a core outcome set
9. Patient burden (number of outcomes and repetition after adding additional outcomes)
10. Cost associated with measuring the core outcome set
11. Lack of accessibility of core outcome sets in different contexts
12. None
13. Other, please specify
14. Which, if any of the following do you think are enablers/facilitators to using core outcome sets in trials? (tick all that apply)
15. Clear understanding of what core outcome sets are
16. Perception that outcomes will be more appropriate for trials
17. Perceived advantages for design of new studies
18. Perceived importance of core outcome sets by trialists/authors/industry
19. Ease of identifying relevant core outcome sets
20. Availability of core outcome set guidelines and resources
21. Recommendations by funders to use core outcome sets
22. Recommendations by professional body to use core outcome sets
23. None
24. Other, please specify
25. What do you think are some of the benefits, if any, of using core outcome sets? (tick all that apply)
26. Standardisation of outcome reporting
27. Reducing outcome reporting bias
28. Reduction in outcome heterogeneity
29. Increase relevance of outcomes to key stakeholders
30. Enhances comparability of findings across trials
31. Increases transparency and openness of research conduct
32. Increases transparency and openness of research reporting
33. Improves quality of trials
34. Can improve quality of evidence syntheses
35. Reduces research waste
36. Enhances patient and public voice in health research
37. Research using core outcome sets can better inform policy and practice
38. None
39. Other, please specify
40. Please let us know any other thoughts or comments you have about core outcome sets
