# Supplementary material for: A survey of knowledge, perceptions and use of core outcome sets among clinical trialists
Source: Trials. 2021 Dec 19;22:937. doi: 10.1186/s13063-021-05891-5 (PMC8684586; doi:10.1186/s13063-021-05891-5)
Supplement: Supplementary file 2 — Additional file 2. Full Participant Country and Health Area Characteristics. [file 13063_2021_5891_MOESM2_ESM.docx]

**Supplementary File 2**

Full Participant Country and Health Area Characteristics

|  | **N (%)** |
| --- | --- |
| **Country** |  |
| United Kingdom | 33 (53.2) |
| Canada | 3 (4.8) |
| Germany | 3 (4.8) |
| Sweden | 3 (4.8) |
| Ireland | 3 (4.8) |
| Saudi Arabia | 3 (4.8) |
| Netherlands | 3 (4.8) |
| India | 2 (3.2) |
| Spain | 2 (3.2) |
| Australia | 1 (1.6) |
| Finland | 1 (1.6) |
| Indonesia | 1 (1.6) |
| Malaysia | 1 (1.6) |
| Martinique | 1 (1.6) |
| Norway | 1 (1.6) |
| Chile | 1 (1.6) |
| **Area of Research** |  |
| Anaesthesia and pain control | 2 (3.2) |
| Blood disorders | 2 (3.2) |
| Cancer | 5 (8.1) |
| Child health | 8 (12.9) |
| Developmental, psychosocial & learning problems | 2 (3.2) |
| Ear, nose and throat | 2 (3.2) |
| Effective practice/health systems | 1 (1.6) |
| Endocrine & metabolic | 3 (4.8) |
| Eyes & vision | 1 (1.6) |
| Gastroenterology | 3 (4.8) |
| Gynaecology | 1 (1.6) |
| Health care of older people | 8 (12.9) |
| Heart & circulation | 5 (8.1) |
| Infectious disease | 5 (8.1) |
| Kidney disease | 1 (1.6) |
| Lungs & airways | 7 (11.3) |
| Mental Health | 8 (12.9) |
| Methodological & Diagnostic | 2 (3.2) |
| Muscle disease | 2 (3.2) |
| Neonatal care | 2 (3.2) |
| Neurology | 8 (12.9) |
| Orthopaedics & trauma | 3 (4.8) |
| Pregnancy & childbirth | 4 (6.5) |
| Public Health | 17 (27.4) |
| Radiology | 2 (3.2) |
| Rehabilitation | 12 (19.4) |
| Rheumatology | 1 (1.6) |
| Skin | 2 (3.2) |
| Tobacco, drugs & alcohol dependence | 1 (1.6) |
| Urology | 1 (1.6) |
| Wounds | 2 (3.2) |
| Homelessness* | 1 (1.6) |
| Social Innovation* | 1 (1.6) |
| Ayurveda Ophthalmology* | 1 (1.6) |
| Social determinant of health* | 1 (1.6) |
| Neuroscience* | 1 (1.6) |
| Psychology* | 1 (1.6) |
| Dietetics & nutrition* | 1 (1.6) |
| Critical care* | 1 (1.6) |
| General & plastic surgery* | 1 (1.6) |
| Obesity* | 1 (1.6) |
| Vector control* | 1 (1.6) |
| Speech & Language therapy* | 1 (1.6) |
| Health information* | 1 (1.6) |
| Pharmaceutical science & hospital pharmacy* | 1 (1.6) |
| Imaging* | 1 (1.6) |

* Health areas self-reported by participants, not listed in the COMET health areas
