# Supplementary material for: A survey of knowledge, perceptions and use of core outcome sets among clinical trialists
Source: Trials. 2021 Dec 19;22:937. doi: 10.1186/s13063-021-05891-5 (PMC8684586; doi:10.1186/s13063-021-05891-5)
Supplement: Supplementary file 3 — Additional file 3. Use of COS in trials by health area. [file 13063_2021_5891_MOESM3_ESM.docx]

**Supplementary File 3**

Use of COS in trials by health area

|  | **N (%)** |
| --- | --- |
| **Health area where COS was used in a trial** |  |
| Cancer | 1 (4.8) |
| Child health | 2 (9.5) |
| Eye & vision | 2 (9.5) |
| Gastroenterology | 1 (4.8) |
| Heart & circulation | 1 (4.8) |
| Lungs & airways | 2 (9.5) |
| Neonatal care | 1 (4.8) |
| Neurology | 4 (19) |
| Orthopaedics & Trauma | 1 (4.8) |
| Pregnancy & childbirth | 1 (4.8) |
| Public health | 1 (4.8) |
| Rehabilitation | 4 (19) |
| Rheumatology | 1 (4.8) |
| Skin | 1 (4.8) |
| Ayurveda ophthalmology* | 1 (4.8) |
| Speech & language therapy* | 1 (4.8) |
| Stroke* | 1 (4.8) |
| Surgery* | 1 (4.8) |

* Health areas self-reported by participants, not COMET health areas.
