# Supplementary material for: A survey of knowledge, perceptions and use of core outcome sets among clinical trialists
Source: Trials. 2021 Dec 19;22:937. doi: 10.1186/s13063-021-05891-5 (PMC8684586; doi:10.1186/s13063-021-05891-5)
Supplement: Supplementary file 4 — Additional file 4. Trialist involved in the development of COS (n=18). [file 13063_2021_5891_MOESM4_ESM.docx]

**Supplementary File 4**

Trialist involved in the development of COS (n=18)

|  | **N (%)** | |
| --- | --- | --- |
|  | **Yes** | **No** |
| **Role in COS development** |  |  |
| Member of COS development team | 9 (50) | 9 (50) |
| COS participant | 9 (50) | 9 (50) |
| **Area of research** |  |  |
| Cancer | 1 (5.6) |  |
| Child health | 2 (11.1) |  |
| Eye & vision | 2 (11.1) |  |
| Gastroenterology | 1 (5.6) |  |
| Health care of older people | 1 (5.6) |  |
| Mental health | 1 (5.6) |  |
| Neurology | 2 (11.1) |  |
| Rehabilitation | 2 (11.1) |  |
| Surgery | 1 (5.6) |  |

Note. Eighteen participants who had previously been involved in development of a core outcome set were asked to answer the questions in the table above.
